# Thyroid Disorders in Central Ghana: The Influence of 20 Years of Iodization

**DOI:** 10.1155/2017/7843972

**Published:** 2017-07-04

**Authors:** Osei Sarfo-Kantanka, Ishmael Kyei, Fred Stephen Sarfo, Eunice Oparebea Ansah

**Affiliations:** ^1^Komfo Anokye Teaching Hospital, Kumasi, Ghana; ^2^Kwame Nkrumah University of Science & Technology, Kumasi, Ghana

## Abstract

**Background:**

Ghana began mandatory iodization of salt in 1996. This study compares the prevalence of thyroid disorders before and after the introduction of iodization.

**Methods:**

This is a retrospective study of thyroid cases from the middle belt of Ghana between 1982 and 2014. To demonstrate a link between iodization and hyperthyroidism and autoimmunity, we compared the prevalence of hyperthyroidism and autoimmune thyroid disorders before and after the iodization programme.

**Results:**

A total of 10,484 (7548 females, 2936 males) cases were recorded. The rate of thyroid cases seen was 343/100,000. Nontoxic nodular goiters (25.7%) and toxic nodular goiters (22.5%) represented the second commonest thyroid disorders recorded. The prevalence of hyperthyroid disorders seen after 1996 was significantly higher than the prevalence seen before the iodization (40.0 versus 21.1%, *p* < 0.001). The prevalence of autoimmune disorders recorded after iodization was significantly higher than that before the iodization programme started (22.3% versus 9.6%, *p* < 0.001).

**Conclusions:**

This study has revealed a significant increase in thyroid admissions in Central Ghana over the decades. A connection between iodine fortification and iodine-induced hyperthyroidism and between iodine fortification and autoimmune thyroiditis has been shown in this study.

## 1. Introduction

Thyroid disorders represent an important public health problem worldwide ranking second to diabetes as the commonest endocrinological disorder seen in adult medical practice and presenting a myriad of devastating consequences if not treated early [1, 2]. The epidemiology and clinical features of thyroid disease are determined by the supply of iodine, an essential element in the synthesis of thyroid hormones [3–5]. Additionally, the presence of both excess and deficient iodine levels can pose adverse health effects [6]. Chronic iodine deficiency results in goiter formation and, if severe, hypothyroidism [7–9]. This can result in severe implications including cretinism, intellectual impairments, increased pregnancy loss, and infant mortality [9–11]. Sub-Saharan Africa has for a long-time been classified as an area of moderate to severe iodine deficiency [12–17]. Thyroid disorders on the continent represent over 25% of the global burden of the disease [18].

The drive by the World Health Organisation and the United Nation International Children Emergency Fund towards the elimination of iodine deficiency disorders through universal iodization of salt and other food products has so far achieved mixed results, particularly on the African continent [14, 19–21]. Whereas persistent wars and famine coupled with difficulties in implementation and regulation of iodine nutrition have eroded some of the gains made towards sufficient iodine intake on parts of the continent [22–24], excessive intake of iodine has been recorded in other areas of the continent [25, 26]. World Health Organisation reports show that adequate or excessive iodine intake borne out of the universal iodization programme has been observed in over 30 countries, some of them on the African continent [27–30]. Aghini-Lombardi et al. in Italy [31, 32], comparing the prevalence of various thyroid disorders in an initial region of severe iodine deficiency before and after the introduction of iodated salt in 1995 in Italy, revealed a reduction in the prevalence of nodular goiter from 46% to 25%, whereas hyperthyroidism and autoantibody levels increased significantly. In Sub-Saharan Africa, a rise in the incidence of thyrotoxicosis was noted by physicians in Zimbabwe and the Democratic Republic of Congo following the introduction of iodized salt in both countries [29, 30].

In Ghana, efforts to reduce endemic iodine deficiency started with the adoption of universal salt iodization programme in the beginning of 1996 [25]. However, the lack of proper institutions to monitor and coordinate activities to ensure a smooth sailing programme devoid of undernutrition and excess iodine nutrition has meant only about 32.5% coverage has been achieved in some areas, while in other areas overexposure of iodine has meant an increase in toxic thyroid disorders [23]. The need therefore has arisen for a comprehensive evaluation of the effect of the 20-year-old universal iodization programme in Central Ghana.

Hospital-based data, although not entirely representative of the community-based setting, provides useful evidence of admission trends while also serving as sentinel reflecting the characteristics of thyroid disorders as what exists in the community. Understanding the temporal changes in thyroid admissions and presentation would therefore be instrumental in predicting the influence of iodization on the epidemiology and clinical presentation of thyroid disease in Ghana. The aim of this study was to assess the trends in demography and clinical characteristics of thyroid admissions from records at a tertiary referral hospital in Kumasi, situated in the middle belt of Ghana between 1982 and 2014.

## 2. Methods

### 2.1. Study Design and Setting

 This is a retrospective study conducted at Komfo Anokye Teaching Hospital, a 1000-bed hospital located at the middle part of Ghana. It is the only tertiary referral hospital serving an estimated population of 10 million people from 6 out of 10 administrative regions of Ghana as well as other neighboring countries. A review of hospital admissions from 1982 to 2014 was performed at the hospital registry. Thyroid outpatient cases seen during the period were obtained from tally cards and the relevant information extracted unto a questionnaire. Among data extracted from the tally cards were age, gender, year of admission, and type of thyroid disorder and these were entered into excel sheets by data entry clerks. Thyroid disease type and diagnosis were classified using the International Classification of Diseases (ICD) codes ICD-9 (from 1983 to 1996) and ICD-10 (from 1997 to 2014). Rates of thyroid cases were expressed as thyroid cases seen per year divided by total number of outpatient cases seen. We considered diagnosis of toxic adenoma, toxic multinodular goiter, and Graves' disease, before and after the introduction of mandatory iodization in Ghana, to demonstrate the possible presence of iodine-induced hyperthyroidism; we considered diagnosis of Graves' disease and Hashimoto's thyroiditis before and after 2005 to demonstrate a link between the administration of iodine and thyroid autoimmunity.

### 2.2. Ethical Approval and Consent

This study was approved by the Committee on Human Research Publication and Ethics of the School of Medical Sciences, Kwame Nkrumah University of Science and Technology, and the Komfo Anokye Teaching Hospital, Kumasi. Patient records/information were anonymized and deidentified prior to analysis.

### 2.3. Statistical Analysis

Means and medians were compared using either the Student *t*-test or Mann–Whitney *U* test for paired comparisons and ANOVA or Kruskal-Wallis test for more than two group comparisons depending on whether continuous variables were parametric or nonparametric. A Poisson regression model was used to examine the temporal trends in the rates of thyroid admissions with categorical year variables. Exact Wilcoxon tests for ordered contingency tables, in the case of categorical characteristics, were used to study time trends. We considered the diagnosis of toxic adenoma/toxic nodular goiter/Graves' disease before and after the introduction of mandatory iodization in Ghana in 1996, to demonstrate the possible presence of iodine-induced hyperthyroidism; we considered diagnosis of Hashimoto's thyroiditis and Graves' disease before and after 1996 to demonstrate a link between the administration of iodine and thyroid autoimmunity. This was done using Pearson Chi-Square analysis. A two-sided *p* value of <0.05 was considered significant in all statistical analysis with no adjustments made for multiple comparisons.

## 3. Results

### 3.1. Thyroid Admissions and Demography

**Figure 1:**
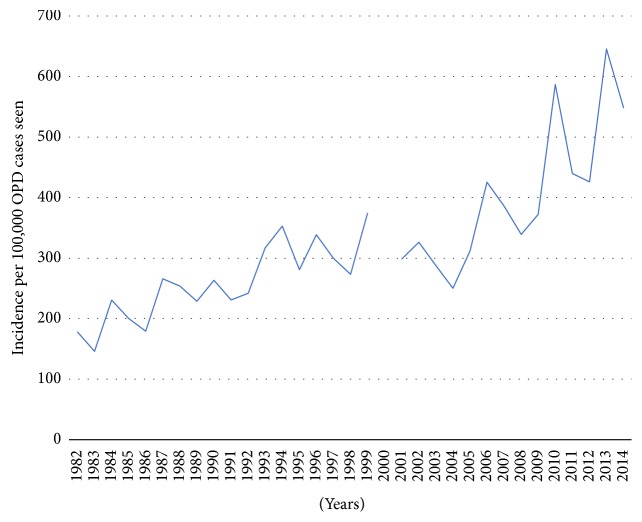
Trends in rates of thyroid admissions in Central Ghana.

Table 1 summarizes the demographic characteristics of this cohort of thyroid admissions. Ten thousand four hundred and eighty-four (10,484) thyroid cases were seen between 1982 and 2014. This was made up of 7584 females and 2936 males with a ratio of 3.5 : 1. The average prevalence rate of thyroid cases seen between 1982 and 2014 was 343/100,000 admissions. As shown in Figure 1, proportions of female thyroid admissions ranged between 58% in 1982 and 81% in 1998. The crude incidence rate of thyroid admissions increased from 146/100,000 patients in 1982 to 426/100,000 patients in 2012. The increase in the rates of thyroid admission was highly significant (*p* < 0.0001).

### 3.2. Characteristics of Thyroid Disorders

Table 2 summarizes the distribution and demographic characteristics of the various thyroid disorders seen over the study period. Nontoxic multinodular goiter represented the commonest thyroid disorder seen over the study period, representing over a quarter of all thyroid admissions. Toxic multinodular goiter represented the second commonest cause of thyroid admissions representing 22.5%. Hypothyroidism, diffuse toxic goiter, nontoxic diffuse goiter, autoimmune thyroiditis, nontoxic adenoma, unspecified thyroiditis, nonspecified/other thyroid disorders, and toxic adenoma represented 13.1%, 12.1%, 6.6%, 6.3%, 5.1%, 5.0%, 3.5%, and 2.1% thyroid admissions, respectively. The median (IQR) age of admission and female: male ratio of patients for the various thyroid disorders were as follows: hypothyroidism: 34 (32.5–52) and 2.2 : 1, nontoxic diffuse goiter: 48 (36–68) and 3.6 : 1, nontoxic thyroid nodule: 34 (32.5–52) and 9.0 : 1, nontoxic multinodular goiter: 40 (30–50) and 3.7 : 1, toxic multinodular goiter: 36 (27–42) and 8.3 : 1, toxic adenoma: 35 (27–42) and 6.1 : 1, Graves' disease: 37 (26–48) and 4.9 : 1, unspecified thyroiditis: 56 (21–70) and 9.0 : 1, Hashimoto's thyroiditis: 23 (31.5–48.5) and 4.1 : 1, and other thyroid disorders: 38 (27–55) and 3.4 : 1. The difference in age of admission between the various thyroid disorders was highly significant (*p* < 0.0001).

### 3.3. Temporal Trends in Thyroid Admissions

#### 3.3.1. Incidence and Demographic Trends

As shown in Table 3, there was an overall progressive increase in the incidence of thyroid cases in both absolute numbers and the rates per overall outpatient cases: 213/100,000 admissions in the 1980s to 538/100,000 admissions in the 2010s (*p* < 0.0001 for linear trend). The trends in the age of thyroid admissions did not differ significantly over the decades. The proportion of female thyroid admissions increased significantly over the decades, increasing from 55.1% in the 1980s to 82.7% in the 2010s (*p* < 0.0001 for trend).

#### 3.3.2. Trends in Thyroid Disorders

There was a significant negative trend in cases of hypothyroidism recorded over the decades, decreasing from 28.2% of thyroid cases in the 1980s to 12.1% in the 2010s (*p* < 0.002 for trend). A significant positive trend was observed for toxic multinodular goiter (3.6% in the 80s versus 46.3% in the 2010s, *p* < 0.001 for trend), nontoxic multinodular goiter (1.4% in the 1980s versus 4.7% in the 2010s, *p* = 0.02 for trend), toxic adenoma (0.3% in the 80s versus 4.2% in the 2010s, *p* < 0.001), diffuse toxic goiter (4.4% in the 80s versus 45.4% in the 2010s, *p* < 0.001 for trend), unspecified thyroiditis (4.8% in the 80s and 28.5% in the 2010s, *p* < 0.0001 for trend), and autoimmune thyroiditis (3.8% in the 1980s versus 10.0% in the 2010s, *p* < 0.0001 for trend). Over the decades, proportion of cases due to nontoxic thyroid nodule, unspecified thyroiditis and other thyroid disorders did not charge significantly.

### 3.4. Thyroid Disorders between 1982–1995 and 1996–2014

#### 3.4.1. Iodine Induces Thyrotoxicosis

As shown in Table 4, diagnosis of toxic multinodular goiter between 1982 and 1995 represented 16.9% of thyroid cases, while from 1996 to 2014 it increased to 24.9% of total thyroid cases (*p* < 0.0001). Diagnosis of Graves' disease between 1982 and 1995 was 5.2%; it increased to 15.1% between 1996 and 2014, *p* < 0.0001. The overall diagnosis of hyperthyroidism/thyrotoxicosis increased significantly between the two time periods from 22.1% of thyroid admission to 40.0% of thyroid admissions. Comparing these results, there was a significant difference between the percentage of thyroid cases due to hyperthyroidism before and after the introduction of mandatory iodization. Prevalence of Hashimoto's autoimmune thyroiditis between 1982 and 1995 was 4.4%; prevalence between 1996 and 2014 increased to 7.2%. The overall diagnosis of autoimmune thyroid disorders over the 2 periods increased from 9.6% to 22.3%. The difference between the 2 periods was statistically significance (*p* < 0.0001) (Table 5).

## 4. Discussion

This is the first study to examine the trajectory of thyroid disorders in Central Ghana before and after introduction of iodized salt in our subregion. We have shown an average incidence of 343/100,000 thyroid cases seen over the study period and an increasing trajectory of thyroid admission in the central belt of Ghana over the last three decades. This is reflected by both an increase in the absolute numbers of thyroid cases and the rate of thyroid cases expressed as the number of thyroid cases per total outpatient cases seen in the hospital over the period. The percentage of medical cases seen over the period due to thyroid disorders increased almost 4-fold over the past three decades in our study. This finding is consistent with findings by Okosieme [10] and Ogbera and Kuku [19]; both recorded increasing incidence rates of thyroid disorders in the subregion. In a retrospective study in Accra, Ghana, in which histological diagnosis of thyroid disorders over a 6-year period from 2004 to 2010 was made, an annual incidence of 185.7 was recorded [33]. This, although lower than the incidence recorded by our study, is mitigated by the cross-sectional nature and the short duration of 6 years under the Accra study. Our study although retrospective was longitudinal with 32 years under study. The trend towards an increase incidence of thyroid disorders worldwide and especially in developing countries like Ghana has largely been explained by the increasing use of sophisticated imaging modalities such as highly sensitive ultrasound for thyroid examinations and third-generation immunoassays in diagnosis [1, 3]. Additionally, Ghana like other countries in the African continent reports nutritional deficiencies including existing selenium deficiency [1, 4, 23]. This has contributed to the erosion of some of the achievement made by the supplementation of iodine [14, 31]. Other endocrine disrupting chemicals such as thiocyanate levels, mostly found in Cassava (a staple food in most African countries) compete with iodine for thyroid hormone synthesis causing a persistence of iodine deficiency disorders [22].

In our study, females represented about 72% of the total thyroid admissions. Our study thus confirms several reports of a clear female gender predisposition to thyroid disease [1, 3, 10]. The increasing trends of thyroid dysfunction in females over the decades recorded in our study may be attributed to the increase of longitudinal trends in the number of autoimmune disorders diagnosed in our study. Autoimmune disorders by themselves have a significant predilection in females [1, 3, 34, 35].

Ghana an initial area with a moderate-severe iodine deficiency instituted mandatory iodization in 1996 [25]. Our results confirm that iodine supplementation especially in regions of moderate-severe iodine deficiency increases the incidence of overt hyperthyroidism from toxic adenoma, toxic multinodular goiter, and Graves' disease. Cerqueira et al. [36] and Pedersen et al. [37] demonstrated this finding by recording an increase in the prevalence of iodine-induced hyperthyroidism after mandatory iodization in Denmark. After universal iodine supplementation came into effect in 1995 in Zimbabwe, the incidence of hyperthyroidism in Zimbabwe increased from 2.8/100,000 to 7.4/100,000 per year [29]. A similar result was found in Zaire [30]. On the contrary, Yang et al. [38] and Teng et al. [39] in their studies failed to demonstrate a connection between iodine fortification and iodine-induced hyperthyroidism. Their studies were conducted in areas of only mild iodine deficiency. Their finding led them to conclude that the hyperthyroidism observed after iodine supplementation was not related to the amount of iodine administrated but was related to the level of iodine deficiency in the area studied, before mandatory iodization.

We have also demonstrated a statistically significant increase in the prevalence of autoimmune thyroid disorders in our hospital after the introduction of mandatory iodization in Ghana. This finding agrees with findings from Pedersen et al. [40], Li et al. [41], and Aghini-Lombardi et al. [31, 32]. Although the exact mechanism by which iodide induces thyroiditis is still unclear, several hypotheses have evolved. These include the presence of excess iodine inducing the production of cytokines and chemokines that can recruit immunocompetent cells to the thyroid, processing of excess iodine in thyroid epithelial cells resulting in elevated levels of oxidative stress and leading to harmful lipid oxidation and thyroid tissue injuries, and iodine incorporation in the protein chain of thyroglobulin augmenting the antigenicity of this molecule. Zimmermann and Andersson [16] on the contrary could not show an increase of thyroid autoantibodies after daily use of iodized salt in Morocco. This contrary finding may be attributed to the strong genetic predisposition to development of autoimmune disease.

The main limitation of this study is the absence of data on thyroid function tests, thyroid autoantibody profile, and lipid profiles. This study is also limited by the lack of detailed demographic and other clinical variables which could explain the increasing incidence of thyroid cases. Lastly, the levels of urinary iodine levels, a measure of iodine adequacy in a patient, were not measured as well as ultrasound measures of thyroid volume. These limitations raise important questions for further studies in helping to elucidate the increasing incidence of thyroid disorders and the changing epidemiology in Ghana. These notwithstanding, we have captured and presented a longitudinal trend in thyroid cases over a 32-year period noting an increasing trend in thyroid cases with a changing epidemiology towards hyperthyroidism and autoimmunity.

It is apparent that the pattern of thyroid disorders is evolving in Central Ghana with increasing iodine sufficiency. Although the primary goal of salt iodination remains the prevention of brain damage due to iodine deficiency, the risks associated with excess iodine replacement in Central Ghana require that attention be paid to increased monitoring of iodization and its effects.

In conclusion, we have shown in this study that the absolute and proportionate numbers of thyroid cases have increased over the past three decades in Central Ghana. There is a progressive increase in the prevalence of hyperthyroid and autoimmune thyroid disorders. These observations should prompt urgent concerted and coordinated efforts at monitoring iodine intake and levels in Central Ghana. While consolidating the present gains, salt iodination programmes must now be subjected to stringent review and monitoring.

## Figures and Tables

**Table 1:** Demography and rates of thyroid disorders seen in Central Ghana from 1982 to 2014.

Year	Number of outpatients seen in a year	Number of thyroid cases seen	Thyroid cases per 100,000 outpatients seen	% female thyroid cases
1982	59594	106	177.9	58.14
1983	57544	82	146.0	65.79
1984	62,474	144	230.5	61.29
1985	67,938	136	200.2	67.24
1986	74,774	134	179.2	68.42
1987	74,768	198	265.9	74.16
1988	74,098	188	253.7	69.15
1989	76,114	174	228.6	59.42
1990	75,168	198	263.4	70.79
1991	73,626	170	230.9	73.85
1992	69,634	166	241.9	76.19
1993	75722	240	316.9	66.67
1994	71404	252	352.9	76.98
1995	70464	198	281.0	62.92
1996	69710	236	338.5	74.62
1997	79464	238	299.5	60.59
1998	73974	202	273.1	81.32
1999	104250	390	374.1	74.62
2000	–	–	–	–
2001	123,618	363	298.5	67.26
2002	127824	417	326.2	77.78
2003	127125	366	287.9	73.91
2004	127,077	318	250.2	75.25
2005	125976	393	312.0	76.58
2006	111,402	474	425.5	76.58
2007	132,138	510	386.0	79.46
2008	131802	447	339.1	64.23
2009	124,971	465	372.1	76.58
2010	134,916	792	587.0	65.58
2011	128,916	567	439.8	71.01
2012	118,308	504	426.0	74.05
2013	115,704	747	645.6	77.84
2014	114,834	630	548.6	71.95

**Table 2:** Thyroid admissions in Central Ghana.

Thyroid disorder	*N*	Median age (IQR)	F : M ratio
Hypothyroidism	1362 (13.1)	34 (32.5–52)	2.2 : 1
Nontoxic diffuse goiter	703 (6.6)	48 (36–68)	3.6 : 1
Nontoxic thyroid nodule	535 (5.1)	34 (32.5–52)	9.0 : 1
Nontoxic multinodular goiter	2694 (25.7)	40 (30–50)	3.7 : 1
Toxic multinodular goiter	2359 (22.5)	36 (27–42)	8.3 : 1
Toxic adenoma	262 (2.5)	35 (27–42)	6.1 : 1
Diffuse toxic goiter	1269 (12.1)	37 (26–48)	4.9 : 1
Thyroiditis unspecified	524 (5.0)	56 (21–76)	2.4 : 1
Autoimmune thyroiditis	660 (6.3)	23 (31.5–48.5)	4.0 : 1
Unspecified thyroid disease	367 (3.5)	38 (27–55)	3.4 : 1

**Table 3:** Temporal trends in incidence and characteristics of thyroid admissions in Central Ghana from 1982 to 2014.

Characteristics		1982–1989	1990–1999	2001–2009	2010–2014	*p* value
Number		1152	2290	3753	3240	<0.0001
Incidence	100,000 admissions	212	229	417	648	<0.0001
Age/years	Mean	43.3 (18.5)	44.8 (18.8)	41.3 (14.1)	43.1 (15.3)	0.7
	Median	43 (30–56)	40 (28–60)	40 (30–50)	42 (31.5–53)	0.06
Women number (%)		638 (54.9)	1456 (66.3)	2931 (78.1)	2631 (81.2)	0.0004
Hypothyroidism	E0 3.9	237 (20.4)	419 (18.3)	188 (5.0)	68 (2.1)	<0.0001
Nontoxic diffuse goiter	E0 4.0	66 (5.7)	46 (2.0)	480 (12.8)	113 (3.5)	0.39
Nontoxic thyroid nodule	E0 4.1	41 (3.5)	121 (5.3)	274 (7.3)	113 (4.4)	0.02
Nontoxic MNG	E0 4.2	595 (51.2)	964 (42.1)	544 (14.5)	363 (11.2)	<0.0001
Toxic MNG	EO 5.2	48 (4.1)	176 (7.7)	1.066 (28.4)	1260 (38.9)	<0.0001
Toxic adenoma		2 (0.3)	32 (1.4)	109 (2.9)	136 (4.2)	<0.0001
Diffuse toxic goiter	E0 5.0	50 (4.3)	137 (6.0)	454 (12.1)	713 (22.0)	<0.0001
Thyroiditis unspecified	EO 6.9	12 (2.1)	30 (2.9)	34 (2.6)	27 (2.5)	<0.12
Autoimmune thyroiditis	E0 6.3	24 (3.8)	66 (5.4)	198 (5.1)	324 (10.0)	<0.00001
Unspecified thyroid/other disease	E0 7.9	40 (3.4)	57 (2.5)	161 (4.3)	62 (1.9)	0.32

**Table 4:** Differences in admission of thyroid disorders before and after iodization in Central Ghana.

Thyroid disorder	1982–1995 *n* = 2385	1996–2014 *n* = 8099	*p* value
Graves' disease, *n* (%)	124 (5.2)	1233 (15.1)	<0.0001
Toxic adenoma/toxic nodular goiter, *n* (%)	403 (16.9)	2017 (24.9)	<0.0001
Total, *n* (%)	527 (22.1)	3240 (40.0)	<0.0001

Graves' disease, *n* (%)	124 (5.2)	1233 (15.1)	<0.00001
Autoimmune thyroiditis, *n* (%)	105 (4.4)	583 (7.2)	<0.00001
Total, *n* (%)	229 (9.6)	1816 (22.3)	<0.0001

**Table 5:** Diagnostic criteria of various thyroid disorders.

Thyroid disease	Diagnostic criteria
*Nodule*	
Single nodule	Normal thyroid volume with a single nodule > 3 mm in diameter
Multiple nodules	Normal thyroid volume with ≥2 nodules > 3 mm in diameter
*Goiter*	
Diffuse	Diffusely increased left and right lobes without nodules on ultrasound
Nodular	Asymmetrical increased left and right lobes or no increase size on ultrasound; irregular dark dense echoes and numerous nodules throughout the thyroid gland
*Hyperthyroidism*	TSH < 0.25 Miu/mL, FT4 > 24 pmol/L, or FT3 > 6.8 pmol/L
*Hypothyroidism*	TSH > 5.0 Miu/mL, TSH < 12.0 pmol/L
*Autoimmune thyroid disease*	
Graves' disease	Hyperthyroidism, diffuse goiter on ultrasound, TPOAb > 34 U/mL, or TRAb >5 U/mL
Hashimoto's thyroiditis	Hypothyroidism, TPOAb > 34 U/mL, or TGAb > 115 U/mL Diffuse goiter on ultrasound without history of thyroid surgery
*Reference ranges*	FT3: 3.7–10.4 pmol/L, FT4: 7.5–21.1 pmol/L, TSH: 0.25–5.0 IU/mL, TPOAb: >5.6 U/L, TRAb > 4.2 U/L
TRAb: thyroid receptor antibody	

This diagnostic criterion has been in place since 2004 and has not changed since its institution. Previously diagnosis was made mainly using clinical examination backed by thyroid function tests.
